# Characterization and pathogenicity evaluation of recombinant novel duck reovirus isolated from Southeast China

**DOI:** 10.3389/fvets.2023.1124999

**Published:** 2023-03-14

**Authors:** Huihu Yang, Wandi Zhang, Meihong Wang, Sheng Yuan, Xuelian Zhang, Feng Wen, Jinyue Guo, Kun Mei, Shujian Huang, Zhili Li

**Affiliations:** ^1^College of Life Science and Engineering, Foshan University, Foshan, Guangdong, China; ^2^Nanyang Vocational College of Agriculture, Nanyang, China

**Keywords:** novel duck reovirus, complete genomes, phylogenetic analysis, re-assortment analyses, pathogenicity

## Abstract

The novel duck reovirus (NDRV) emerged in southeast China in 2005. The virus causes severe liver and spleen hemorrhage and necrosis in various duck species, bringing serious harm to waterfowl farming. In this study, three strains of NDRV designated as NDRV-ZSS-FJ20, NDRV-LRS-GD20, and NDRV-FJ19 were isolated from diseased Muscovy ducks in Guangdong and Fujian provinces. Pairwise sequence comparisons revealed that the three strains were closely related to NDRV, with nucleotide sequence identities for 10 genomic fragments ranging between 84.8 and 99.8%. In contrast, the nucleotide sequences of the three strains were only 38.9–80.9% similar to the chicken-origin reovirus and only 37.6–98.9% similar to the classical waterfowl-origin reovirus. Similarly, phylogenetic analysis revealed that the three strains clustered together with NDRV and were significantly different from classical waterfowl-origin reovirus and chicken-origin reovirus. In addition, the analyses showed that the L1 segment of the NDRV-FJ19 strain was a recombinant of 03G and J18 strains. Experimental reproduction of the disease showed that the NDRV-FJ19 strain was pathogenic to both ducks and chickens and could lead to symptoms of hemorrhage and necrosis in the liver and spleen. This was somewhat different from previous reports that NDRV is less pathogenic to chickens. In conclusion, we speculated that the NDRV-FJ19 causing duck liver and spleen necrosis is a new variant of a duck orthoreovirus that is significantly different in pathogenicity from any previously reported waterfowl-origin orthoreovirus.

## 1. Introduction

Avian orthoreoviruses (ARVs) are members of the *Orthoreovirus* genus, part of the *Reoviridae* family (1). These viruses are double-stranded RNA viruses with a complete virus particle diameter of 70–80 nm, no envelope, and an icosahedral double-shell structure (2). The complete genome is made up of 10 segments that can be classified into three groups based on sodium dodecyl sulfate-polyacrylamide gel electrophoresis (SDS-PAGE) mobility: λ, μ, and σ proteins. These are encoded by the large segments L (L1, L2, and L3), the middle segments M (M1, M2, and M3), and the subsegments S (S1, S2, S3, and S4), respectively (3, 4). Each segment's first seven bases located at the 5' end (5'-GCUUUUU) and the last five located at the 3' end (UCAUC-3') are highly conserved (5). Based on the genome sequence, host, and pathological changes, ARVs are classified as chicken-origin reovirus (chicken ARV), classical waterfowl-origin reovirus, and novel duck reovirus (NDRV) (5, 6).

Chicken ARV was first isolated in 1954 from broiler chickens suffering from chronic respiratory diseases (7). Arthritis, tenosynovitis, and intestinal and respiratory diseases were all clinical manifestations of chicken ARV infection, and infection was common in chickens in China, with a positive rate of 27% for antibodies detected in chicken serum samples (8). Classical waterfowl-origin reovirus was designated as Muscovy duck reovirus (MDRV) and was subsequently isolated from Muscovy ducks in France in 1972 and introduced into China in 1997 (9). The primary target of infection was Muscovy ducklings between 10 and 20 days of age, and the white necrosis spots in the liver and spleen were the characteristic pathological changes (10, 11). The mortality rate reached 30% (11, 12).

In 2002, new viral infections emerged in Muscovy ducks, Peking ducks, and many others in southeast China (13, 14). The disease was common in ducklings aged 5–10 days, with an incidence rate of 10–40% and a fatality rate of 15–50%; the younger the age of the impacted ducks, the greater the severity and fatality rate (15, 16). Immunosuppression, slow development, severe diarrhea, hemorrhagic patches, and necrosis of the liver and spleen of ducklings were the most common symptoms of this viral infection (15). In addition, the virus causing this disease had more hosts and more significant infection rate than MDRV, and it was pathogenic to a wide range of ducks (12, 15, 17). Therefore, the virus was named NDRV (13). Phylogenetic analysis of nucleotide sequences based on S1 and S3 segments also showed that NDRV was significantly different from MDRV and chicken ARV (5, 13).

Because of its high morbidity and mortality and the susceptibility of many species of ducks, NDRV has become one of the most important infectious diseases in duck breeding in China (17, 18). Therefore, continuous surveillance is required to obtain information concerning endemic and emerging wild-type NDRV strains in waterfowl in order to develop effective prevention strategies. In this study, three strains of NDRV designated as NDRV-FJ19, NDRV-ZSS-FJ20, and NDRV-LRS-GD20 were isolated from Guangdong and Fujian provinces between 2019 and 2020. The whole genomes of the three strains were cloned, sequenced, and analyzed, and the pathogenicity of NDRV-FJ19 to ducks and chicks was investigated. The experimental data of these three strains may provide a new theoretical foundation for NDRV monitoring, prevention, and control.

## 2. Materials and methods

### 2.1. Cell lines and experimental animals

A Vero African green monkey kidney cell line (Vero cells), a Baby Hamster Syrian Kidney line (BHK-21 cells), and Leghorn Male-chicken Hepatocellular-carcinoma cells (LMH cells) were provided by the College of Life Science and Engineering. Duck parvovirus virus (DPV), Muscovy duck reovirus (MDRV), and duck Tembusu virus (DTMUV) were stored in our laboratory. One-day-old healthy ducklings and one-day-old SPF chicks were provided by Guangzhou South China Agricultural University Biological Drug Co., Ltd.

### 2.2. Primer design

Primer Premier 5.0 was used to design primers, and the details of primers are shown in Table 1.

**Table 1:** Primers used in this study.

**Virus**	**Primers**	**Oligonucleotide sequence (5' → 3')**	**Product size (bp)**	**GenBank accession numbers**
Novel duck reovirus	NDRV-F	ATCAGCGTGGTTTTGAGTAT	263	KF154111
	NDRV-R	GAGAGACCATCGACAATCAT		
Duck parvovirus virus	DPV-F	ACAGGCGGAACAGATAAT	441	MF962899
	DPV-R	GAGATTCGGAGAAGGATG		
Muscovy duck reovirus	MDRV-F	GCACTCTGGATCCAGTAC	438	KF306091
	MDRV-R	CAATGGAGAAGCGAAC		
Duck Tembusu virus	TMUV-F	ACAGATGCTCGACGGACT	296	NC015843
	TMUV-R	ACCAGCAGTCTATGTCTTCAG		

### 2.3. Sample collection

Three Muscovy duck farms in China's Fujian and Guangdong provinces had cases of hemorrhagic patches and necrosis of the liver and spleen in Muscovy ducklings. To explore the etiology of the disease, liver and spleen samples from the afflicted ducks were obtained in August 2019 and June and August 2020, mixed with phosphate buffer saline (PBS) (pH 7.2) at a ratio of 1:3, and then freeze-thawed three times.

### 2.4. Virus isolation and identification

The supernatant was filtered through a 0.22-μm filter membrane and inoculated on a monolayer of Vero cells after centrifugation at 12 000 r/min for 15 min (17). The viral inoculant was discharged after 1 h of adsorption. The cells were cultured for 5–7 days at 37°C with 5% CO_2_ in DMEM with 1% FBS (Gibco, Shanghai, China). When cytopathic effects (CPEs) of >75% were observed, viral cultures were collected and sub-cultured until a stable CPE could be obtained (19). Then, the virus solution was diluted by gradient and plaque purification technology for virus purification. Finally, the cell cultures were frozen and thawed three times and stored for later use (20).

The viral RNAs of cell cultures were extracted according to the instructions of a virus nucleic acid extraction kit (Magen, Guangzhou, China) and stored at −80°C. The reverse transcription-polymerase chain reaction (RT-PCR) with a PrimeScript^TM^ One Step RT-PCR Kit (Takara, Dalian, China) was used to identify NDRV, Duck parvovirus virus (DPV), MDRV, and Duck Tembusu virus (DTMUV) viral RNAs. The purified virus was used to measure the TCID_50_, which was computed using the Reed-Muench technique (21). The NDRV-FJ19 strain (MOI = 0.1) was inoculated in three cell lines (Vero cells, BHK-21 cells, and LMH cells), and the viral cultures were harvested at 12, 24, 36, 48, 60, 72, 84, 96, 108, and 120 h and used to measure the TCID_50_ and plot growth curves through data measurement.

### 2.5. Experimental reproduction of the disease with the isolated virus

To validate the pathogenicity and infectivity of the NDRV-FJ19 strain, we conducted an experimental reproduction on 30 one-day-old healthy ducklings and 30 one-day-old SPF chicks. Each duckling and chick were injected intramuscularly with 0.5 mL of the NDRV-FJ19 virus (TCID_50_ = 10^−4.25^/0.1 mL). As a control, 30 ducklings and 30 chicks were inoculated with PBS in the same manner. Ducklings and chicks were observed daily, and symptoms were recorded. Twelve ducklings (six control and six infected) and 12 chicks (six control and six infected) were killed on the third, sixth, ninth, 12th, and 15th days after the start of infection (22). Liver and spleen tissues were examined to determine if pathological changes had occurred, and then, tissues were collected for pathological examination and virus re-isolation. The tissue samples were fixed in 10% formaldehyde solution for 24 h, and embedded in paraffin. Tissue sections were prepared from the paraffin blocks and stained with hematoxylin and eosin (HE) for microscopic observation. Ultimately, the virus was confirmed with RT-PCR analysis and gene sequencing. (All the animal infection experiments were reviewed and approved by Animal Protection and Ethics Committee and Use Committee of Foshan University).

### 2.6. Complete genome segment amplification and sequencing

The stored viral RNA samples were collected, and genomes of all segments were cloned using RT-PCR with a PrimeScript^TM^ One Step RT-PCR Kit (Oligonucleotide primers in Additional File 1) (5). The reaction program was as follows: 50°C for 30 min, 94°C for 3 min, then 35 cycles at 94°C for 30 s, 50–60°C for 30 s, extension at 72°C for 1 min, and then a further extension for another 10 min at 72°C. The RT-PCR products were electrophorized on 1% agarose gels, and the results were viewed and recorded using a gel imaging analyzer (Tianneng Technology Co., Ltd., Shanghai, China). A Gel Extraction Kit (Omega Bio-Tek, Beijing, China) was used in the purification of RT-PCR products, and the recovered product was cloned to the pMD18-T vector (TaKaRa Biotechnology Company, Dalian, China) and transformed into *E.coli* DH5α competent cells (TaKaRa). The positive bacterial solution was sent to Sangon Biotech (Guangzhou, China).

### 2.7. Sequence comparisons and phylogenetic analyses

The nucleotide sequence was spliced and translated with DNASTAR Lasergene 12 Core Suite. Sequence similarity was evaluated by using BLAST in GenBank (http://blast.ncbi.nlm.nih.gov/Blast.cgi). ORFfinder (http://www.ncbi.nlm.nih.gov/gorf/gorf.html) was used to predict the virus' open reading frames (ORFs) in sequences. Sequences were aligned by using the Megalign V7.0 and MEGA V5.0 programs. MEGA V5.0's Maximum Likelihood (ML) and bootstrap support values computed for 1000 repetitions were used to construct a phylogenetic tree. Mammalian reovirus (MRV) was used as an outgroup.

### 2.8. Recombination detection

All segment sequences of the three strains were screened for recombination by using the RDP, GENECONV, and BootScan methods in Recombination Detection Program, version 4 (RDP4). Sequence recombination was validated using at least two methods. In addition, the parent strains of the recombinants were visualized by using SimPlot Version 3.5.1, with a window size of 200 bp and a step size of 20 bp.

## 3. Results

### 3.1. Virus isolation and purification

**Figure 1:**
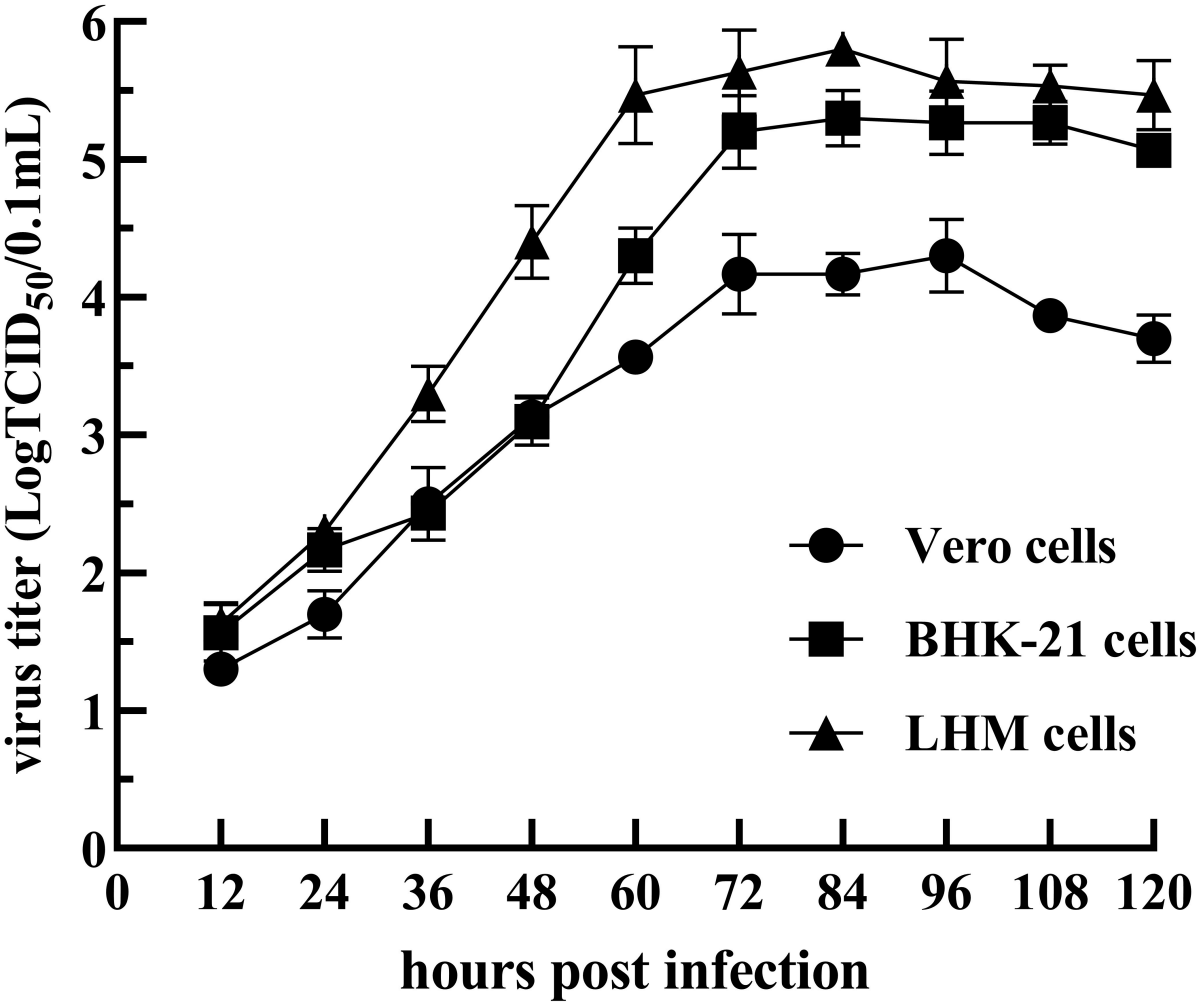
Growth curve of the NDRV-FJ19 strain in Vero, BHK-21, and LMH cells.

**Figure 2:**
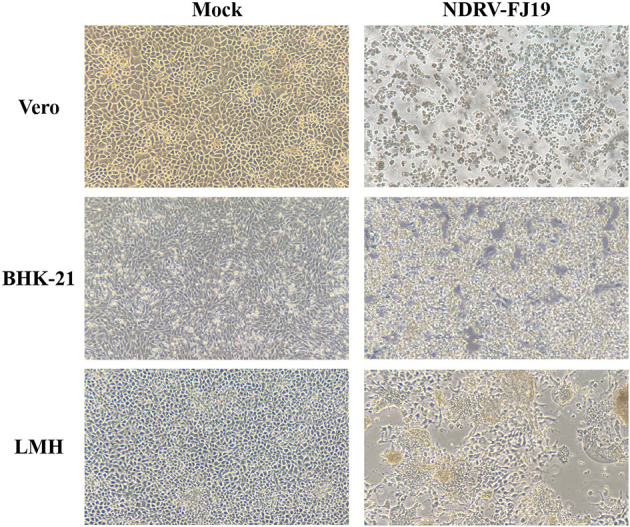
CPE of NDRV-FJ19 strain at 72 h post infection in the three cell lines.

Three liver and spleen samples were inoculated into Vero cells for five generations, and all were capable of causing visible CPE in Vero cells within 3–5 days, leading to cell fusion and syncytial formation. In the RT-PCR analysis, NDRV was positive, while DPV, MDRV, and DTMUV were negative. Three isolates, NDRV-FJ19, NDRV-ZSS-FJ20, and NDRV-LRS-GD20, were successfully isolated and purified using plaque purification technology. Table 2 lists the TCID_50_ values of the three viruses on Vero cells. The growth curves of the NDRV-FJ19 strain in three cell lines are shown in Figure 1, and the corresponding cytopathic effects are shown in Figure 2. The virus titer of NDRV-FJ19 reached the highest level at 72 h and remained there for a period of time. (Details of data were provided in Additional File 2).

**Table 2:** Information on three novel duck reovirus strains.

**Strain**	**Host**	**TCID_50_/0.1 mL**	**Isolation of time**	**Province**	**GenBank accession numbers**
NDRV-ZSS-FJ20	Muscovy duck	10^−4.0^	2020/08	Fujian	OM930755- OM930764
NDRV-LRS-GD20	Muscovy duck	10^−3.67^	2020/06	Guangdong	OM930745- OM930754
NDRV-FJ19	Muscovy duck	10^−4.25^	2019/08	Fujian	OM930735- OM930744

### 3.2. Experimental reproduction of infection

**Figure 3:**
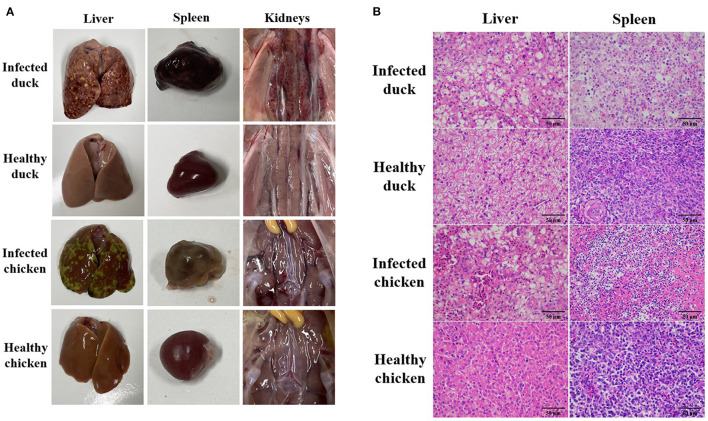
Pathological changes in ducks and chickens artificially infected with NDRV-FJ19 strain. **(A)** Gross lesions of duck and chicken. Hemorrhage and necrosis of the liver and the spleen, and severe hemorrhage and swelling of kidneys in infection group. **(B)** Histopathological changes of duck and chicken. Scale bar: 50 μm. In infection group, HE-stained liver section showing hemorrhage in liver tissue and swelling of hepatocytes. HE-stained spleen section showing diffuse hemorrhage and necrosis of splenocytes; many splenic lymphocytes have atrophic nuclei.

After the infection with the NDRV-FJ19 strain, the infected group of ducklings experienced a significant peak in mortality at 4–7 days (mortality rate rising to 46.7%). The infected group of chicks experienced a significant peak in mortality at 2–5 days (mortality rate rising to 33.3%). Symptoms of hemorrhage and necrosis in livers and spleens (Figure 3A) and hemorrhage and swelling of kidneys (Figure 3A) were observed, similar to symptoms acquired by naturally infected ducks. Histopathological changes in the liver and spleen were observed (Figure 3B). Finally, the virus was successfully recovered from infected or dead birds and confirmed by RT-PCR and sequencing. The virus was detected from the heart, lungs, liver, spleen, kidneys, small intestine, and bursae of infected animals.

### 3.3. Analysis of the isolates' genomic segments

Genome cloning and sequencing yielded the entire genome sequences of the three isolates, and these were validated as NDRV by BLAST in the NCBI database8 (http://blast.ncbi.nlm.nih.gov/Blast.cgi). The complete sequences of the NDRV-FJ19, NDRV-LRS-GD20, and NDRV-ZSS-FJ20 genomes were deposited into GenBank under accession numbers OM930735-OM930744, OM930745-OM930754, and OM930755-OM930764, respectively. Table 3 shows the particular details of genomic sequences. The genomes of the three isolates were arranged similarly to *Orthoreovirus*, with the entire genomes of 23,418 bp split into 10 segments: L1 (3959 bp), L2 (3830 bp), L3 (3907 bp), M1 (2283 bp), M2 (2158 bp), M3 (1996 bp), S1 (1568 bp), S2 (1324 bp), S3 (1202 bp), and S4 (1191 bp). ORFfinder analysis revealed that the three isolates' S1 genes were triscistrons, with three ORFs encoding P10 (20-313 bp), P18 (273-761 bp), and σC (571-1536 bp). With the exception of the S1 segment, all NDRV segments contained only one ORF that sequentially encoded proteins λA, λB, λC, μA, μB, μNS, σA, σB, and σNS.

**Table 3:** General genome features of the three novel duck reovirus strains.

**Genome segment**	**Length of nucleotide sequence (bp)**			**ORF location**	**Protein size (aa)**	**Encoded protein**
	**NDRV-ZSS-FJ20**	**NDRV-LRS-GD20**	**NDRV-FJ19**			
L1	3,959	3,959	3,959	22–3,903	1,293	λA
L2	3,830	3,830	3,830	15–3,794	1,259	λB
L3	3,907	3,907	3,907	13–3,870	1,285	λC
M1	2,283	2,283	2,283	13–2,211	732	μA
M2	2,158	2,158	2,158	30–2,057	675	μB
M3	1,996	1,996	1,996	25–1,932	635	μNS
S1	1,568	1,568	1,568	20–313 273–761 571–1,536	97 162 321	P10 P18 σC
S2	1,324	1,324	1,324	16–1,266	416	σA
S3	1,202	1,202	1,202	31–1,134	367	σB
S4	1,191	1,191	1,191	24–1,127	367	σNS

### 3.4. Pairwise sequence comparisons

To clarify the relationship between NDRV-ZSS-FJ20, NDRV-LRS-GD20, and NDRV-FJ19 and their identity with other ARVs, 23 published reference strains of *Orthoreovirus* from GenBank (Additional File 3) were selected, with 10 strains of NDRV, four strains of MDRV, six strains of chicken ARV, and three strains of MRV. The results of sequence comparison (Additional File 4) revealed that the three NDRV strains in this experiment shared a high degree of similarity in nucleotide (nt, 86.7–99.1%) and amino acid sequences (aa, 97.0–99.9%). The three isolates significantly differed in their similarity to MDRV and chicken ARV and had the highest sequence identity with NDRV (nt, 86.5–99.9%; aa, 94.5–99.9%). NDRV-ZSS-FJ20 and NDRV-LRS-GD20 had the highest sequence identity with recently isolated SH12, DH13, and HN5d (nt, 94.2–99.9%; aa, 96.9–99.9%), while NDRV-FJ19 had the highest sequence identity with NP03 (nt, 95.2–99.9%; aa, 97.5–99.9%). In the nucleotide sequence encoding of σC, NDRV-ZSS-FJ20, NDRV-LRS-GD20, and NDRV-FJ19 shared the highest sequence identity with DH13, SH12, NP03, and SD-12 (nt, 95.1–99.7%; aa, 95.5–98.8%). The nucleotide sequence similarity between the three isolates and MDRV was 37.6–95.0%, among which σA encoding genes had the highest homology (nt, 88.4–95.0%) and σC encoding genes had the lowest homology (nt, 37.6–39.5%). The similarity of the three isolates with chicken ARV varied from 38.9 to 80.9%, with the most significant identity with the σA encoding genes (nt, 77.2–80.9%) and the lowest with the σC encoding genes (nt, 38.9–40.5%). The nucleotide and protein sequence of σC encoding genes differed the most between NDRV, MDRV, and chicken ARV. The λA, λB, σA, and σNS encoding genes had the least divergence, and nucleotide sequence homology was >76.8%, indicating that these four segments of avian reoviruses remained conserved.

### 3.5. Phylogenetic analyses

**Figure 4:**
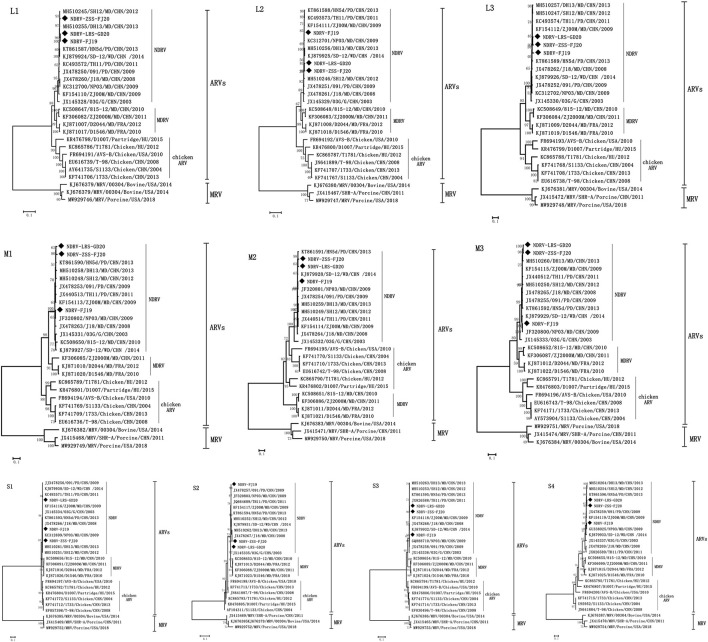
Phylogenetic trees constructed based on the nucleotide sequences of the L, M, and S genome segments of different reoviruses. The three strains used in this experiment are shown as solid diamonds: NDRV-ZSS-FJ20, NDRV-LRS-GD20, and NDRV-FJ19. The scale is equal to the genetic distance between two individuals.

The ML method in the MEGA V5.0 program was used to create phylogenetic trees based on nucleotide sequences of 10 genome segments to explore the phylogenetic relationships between the three viruses obtained in this experiment and with NDRV, MDRV, and chicken ARV. As illustrated in Figure 4, the reference sequences were clearly split into four monophyletic groups: the NDRV subgroup, the MDRV subgroup, the chicken ARV subgroup, and the MRV subgroup. Meanwhile, all waterfowl- and chicken-origin isolates also formed two separate host-associated clades (except M2 and S2 segments). The three strains of NDRV-ZSS-FJ20, NDRV-FJ19, and NDRV-LRS-GD20 and other NDRV isolates (SH12, DH13, 091, TH11, NP03, J18, ZJ00M, and HN5d) formed a monophyletic branch distinct from MDRV and chicken ARV. Therefore, the three strains in this experiment can be confirmed as NDRV. NDRV-ZSS-FJ20 and NDRV-LRS-GD20 had a close relationship (except S1 segments) and were most closely associated with the DH13 and SH12 strains in the phylogenetic trees based on the L1, L2, M3, and S2 segments; in addition, they were the closest relatives to ZJ00M strains in the phylogenetic trees based on the L3, S1, and S3 segments and were the closest relatives to HN5d strains in the phylogenetic trees based on the M1, M2, and S4 segments. NDRV-FJ19 was the most closely related strain to NP03 in L2, M1, M2, M3, S1, and S3 gene segments isolated in 2009 in Fujian, China. This suggests that NP03 played an important role in the formation of NDRV-FJ19 virus strain.

### 3.6. Recombination analyses

**Figure 5:**
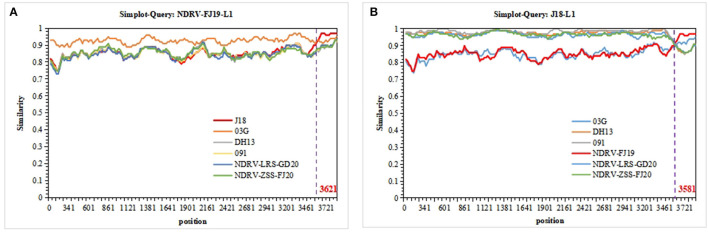
Recombination analyses of the segment sequences of three strains. NDRVs and others were screened using RDP4 and visualized using SimPlot 3.5.1. **(A)** Recombination analysis of the L1 segments of NDRV-FJ19. **(B)** Recombination analysis of the L1 segment of J18.

The sequences of 10 segments of the three strains were evaluated for the presence of recombination using RDP4 and SimPlot. Using the sequences of J18, 03G, DH13, and 091 as the parental sequences, a recombination event that was supported by the RDP and similarity plot analyses was found in the L1 sequence of the NDRV-FJ19 strain. According to the similarity plot analysis, the recombination breakpoint occurred at position 3,621 of the sequence alignment (Figure 5A). Therefore, it is reasonable to speculate that the L1 sequence of the NDRV-FJ19 strain was reconstituted by 03G and J18 strains. In addition, another recombination event was found in the L1 sequence of the J18 strain, similar to the NDRV-FJ19 strain, and the recombination breakpoint occurred at position 3,581 of the sequence alignment (Figure 5B). Apart from the above, the recombination events in the S2 segments of ZJ00M, J18, HN5d, SH12, and DH13 and in the M2 segments of NP03 and D1007 were detected, but these recombination events were not effectively statistically supported by RDP or similarity plot analysis.

## 4. Discussion

Since the outbreak of the first epidemic disease characterized by irregular necrosis of duck liver and spleen in 2005, the industry has suffered massive economic losses (23). As a multi-segment double-stranded RNA virus, NDRV is prone to recombination during transmission, leading to the emergence of new variants (5). Therefore, it is necessary to monitor the sequence of NDRV. In this study, three strains of NDRV were isolated, and the sequence and pathogenicity were preliminarily analyzed.

The sequencing analysis of the three strains revealed that the full genomes were 23,418 bp in length, and the length of each segment was almost identical to that of the reported matching NDRV genome segments (5, 13). In terms of genomic structure, three strains of NDRV were more comparable to chicken ARV and were considerably distinct from MDRV S-class segments due to the different locations of the proteins encoded. The S1 segment was polycistronic with three overlapping ORFs, similar to chicken ARV. NDRV S1–S4 segments encoded P10, P18, σC, σA, σB, and σNS proteins, while MDRV S1–S4 segments encoded σA, σB, σNS, P10, and σC proteins (23). The S4 segment of MDRV was a dicistron that did not produce the P18 protein; this was the most significant change in genomic structure between NDRV and MDRV (24).

Many previous studies have shown that the σC protein (16, 25, 26), as the capsid structural protein of virions, is the most important antigenic protein of viruses, meaning that it primarily determines the pathogenicity of viruses. Therefore, the σC encoding genes can be used as genetic markers for ARV differentiation and classification. The similarity of nucleotide sequence encoding σC between NDRV and MDRV was 37.6–39.5% and that between NDRV and chicken ARV was 38.9–40.5%. The current international classification and naming of NDRV are unclear. As a result, we believe that NDRV should be categorized as a unique waterfowl-origin reovirus distinct from chicken ARV and MDRV. Furthermore, the nucleotide sequences of the L1, L2, and M2 gene segments of a goose-origin (03G) NGRV and a duck-origin NDRV were considerably different. Whether the nucleotide sequences of goose-origin NGRV and duck-origin NDRV differ because of host origin has not been confirmed. This could not be statistically analyzed, since there are few reported cases of goose-origin NGRV. This perspective should be investigated further.

Looking at the structure of the phylogenetic trees, waterfowl reovirus-origin NDRV and MDRV constituted a substantial clade independent of chicken ARV, suggesting that most chicken ARVs and waterfowl-origin reoviruses (WRVs) have clearly evolved to be host specific. However, NDRV and chicken ARV were on the same branch in the M2 segment, independent of MDRV. This unusual occurrence was not seen in the other segments. We assumed that this was due to recombination between WRVs and chicken ARVs. Recombination events in segments of the genome are very common in ARVs (5). In research reports of recent years, most of the orthoreovirus variants that have emerged are generated by viral recombination, for example, the SDPY-ARV and N-DRV-XT18 strains isolated in Shandong, China (17, 27). These strains were formed by recombination between WRVs or between WRVs and chicken ARVs; this not only resulted in the strains becoming more virulent but also widened the range of hosts. At present, the mechanism and extent of Reoviridae RNA recombination are undefined (1, 2, 28). Some scholars have found that reovirus accumulates defective gene segments with internal deletions during the passage process, and carries out sequence-directed recombination at different sites (28, 29). The titers of these recombinant viruses were significantly increased on the cells8 (29). This may be the reason why the strain NDRV-FJ19 strain was more pathogenic. Therefore, recombination events of ARVs deserve close attention. Strains isolated from different regions of the country were in the same lineage. The isolates from France (D1546, D2044) in the MDRV branch showed significant homology (94.5–98.6%) with the isolates from China, indicating the existence of a regional reovirus difference that should be confirmed further.

From the results of experimental reproduction of infection, we found that NDRV-FJ19 strains cause serious damage to the liver and spleen of ducks and chickens, especially the severe bleeding and necrosis of spleen, necrosis of splenocytes, and lymphocyte depletion. The spleen, as an important immune organ of poultry, plays a major role in the body's immune function (30). However, NDRV infection reduces the number of lymphocytes in the spleen and leads to immunosuppression (27, 31). Therefore, the disease should be treated as early as possible; otherwise, it will become more serious. In addition, it has been shown in previous studies that waterfowl-derived orthoreovirus tends to infect only waterfowl or is weakly infectious to chickens (12, 15, 31), while chicken ARV infects only chickens (32, 33). Although NDRV-FJ19 is a waterfowl-derived orthoreovirus, it is highly pathogenic to chickens, a phenomenon previously unobserved. Therefore, we speculated that this virus is a new variant of a duck orthoreovirus, and we further verified the tendency of NDRV to adapt to multiple hosts.

## 5. Conclusion

In this investigation, three strains of NDRV were isolated from Muscovy ducks in Guangdong and Fujian provinces, and the whole genome sequences were identified. The three strains were closely related to orthoreovirus isolates derived from ducks and geese, with nucleotide sequence identities for 10 genomic fragments ranging between 37.6 and 99.8%. In contrast, the nucleotide sequences of genomic fragments from the three strains were only 38.9–80.9% similar to a chicken orthoreovirus isolate. Meanwhile, the strains had stronger pathogenic properties than other members in the orthoreovirus genus, suggesting that the waterfowl-derived orthoreovirus has mutated in the direction of increased virulence.

## Data availability statement

The datasets presented in this study can be found in online repositories. The names of the repository/repositories and accession number(s) can be found in the article/Supplementary material.

## Ethics statement

The animal study was reviewed and approved by Animal Protection and Ethics Committee and Use Committee of Foshan University.

## Author contributions

HY, MW, and WZ designed the study. FW, JG, KM, SH, and ZL analyzed the data together. HY and WZ wrote the initial draft of the manuscript. SH and ZL revised the manuscript. All authors performed the experiments and reviewed the manuscript.

## References

[1] Zhang J Li T Wang W Xie Q Wan Z Qin A. Isolation and molecular characteristics of a novel recombinant avian orthoreovirus from chickens in China. Front Vet Sci. (2021) 8:771755. 10.3389/fvets.2021.771755 34950724 PMC8688761

[2] Yan T Guo L Jiang X Wang H Yao Z Zhu S. Discovery of a novel recombinant avian orthoreovirus in China. Vet Microbiol. (2021) 260:109094. 10.1016/j.vetmic.2021.109094 34271302

[3] Noh J Lee D Lim T Lee J Day J Song CJAov. Isolation and genomic characterization of a novel avian orthoreovirus strain in Korea, 2014. Arch Virol. (2018) 163:1307–16. 10.1007/s00705-017-3667-8 29392490

[4] Niu X Tian J Yang J Jiang X Wang H Tang Y. Complete genome sequence of a novel avian orthoreovirus isolated from gosling, China. Arch Virol. (2018) 163:3463–6. 10.1007/s00705-018-4035-z 30209584

[5] Zhang XL Shao JW Li XW Mei MM Guo JY Li WF. Molecular characterization of two novel reoviruses isolated from Muscovy ducklings in Guangdong, China. BMC Vet Res. (2019) 15:143. 10.1186/s12917-019-1877-x 31077188 PMC6511161

[6] Farkas S Varga-Kugler R Ihász K Marton S Gál J Palya V. Genomic characterization of avian and neoavian orthoreoviruses detected in pheasants. Virus Res. (2021) 297:198349. 10.1016/j.virusres.2021.198349 33631220

[7] Heggen-Peay C Qureshi M Edens F Sherry B Wakenell P O'Connell P. Isolation of a reovirus from poult enteritis and mortality syndrome and its pathogenicity in turkey poults. Avian Dis. (2002) 46:32–47. 10.1637/0005-2086(2002)046[0032:IOARFP]2.0.CO;2 11922348

[8] Mase M Gotou M Inoue D Masuda T Watanabe S Iseki H. Genetic analysis of avian reovirus isolated from chickens in Japan. Avian Dis. (2021) 65:346–50. 10.1637/0005-2086-65.3.346 34427406

[9] Li Y Yin X Chen X Li X Li J Liu C. Antigenic analysis monoclonal antibodies against different epitopes of σB protein of Muscovy duck reovirus. Virus Res. (2012) 163:546–51. 10.1016/j.virusres.2011.12.006 22197425

[10] Wozniakowski G Samorek-Salamonowicz E. Gaweł AJPjovs. Occurrence of reovirus infection in Muscovy ducks (Cairina moschata) in south western Poland. Pol J Vet Sci. (2014) 17:299–305. 10.2478/pjvs-2014-0041 24988856

[11] Chen S Lin F Chen S Hu Q Cheng X Jiang B. Development of a live attenuated vaccine against Muscovy duck reovirus infection. Vaccine. (2018) 36:8001–7. 10.1016/j.vaccine.2018.10.102 30420117

[12] Yun T Yu B Ni Z Ye W Chen L Hua J. Isolation and genomic characterization of a classical Muscovy duck reovirus isolated in Zhejiang, China. Infect Genet Evol. (2013) 20:444–53. 10.1016/j.meegid.2013.10.004 24140560

[13] Yun T Yu B Ni Z Ye W Chen L Hua J. Genomic characteristics of a novel reovirus from Muscovy duckling in China. Vet Microbiol. (2014) 168:261–71. 10.1016/j.vetmic.2013.11.005 24355531

[14] Chen Z Zhu Y Li C Liu G. Outbreak-associated novel duck Reovirus, China, 2011. Emerg Infect Dis. (2012) 18:1209–11. 10.3201/eid1807.120190 22709408 PMC3376814

[15] Li N Hong T Wang Y Wang Y Yu K Cai Y. The pathogenicity of novel duck reovirus in Cherry Valley ducks. Vet Microbiol. (2016) 192:181–5. 10.1016/j.vetmic.2016.07.015 27527781

[16] Zheng X Wang D Ning K Liang T Wang M Jiang M. A duck reovirus variant with a unique deletion in the sigma C gene exhibiting high pathogenicity in Pekin ducklings. Virus Res. (2016) 215:37–41. 10.1016/j.virusres.2016.01.020 26829009

[17] Wang S Lin F Cheng X Wang J Zhu X Xiao S. The genomic constellation of a novel duck reovirus strain associated with hemorrhagic necrotizing hepatitis and splenitis in Muscovy ducklings in Fujian, China. Mol Cell Probes. (2020) 53:101604. 10.1016/j.mcp.2020.101604 32502523

[18] Yan H Xu G Zhu Y Xie Z Zhang R Jiang S. Isolation and characterization of a naturally attenuated novel duck reovirus strain as a live vaccine candidate. Vet Microbiol. (2021) 261:109214. 10.1016/j.vetmic.2021.109214 34461358

[19] Yang J Tian J Chen L Tang Y Diao Y. Isolation and genomic characterization of a novel chicken-orign orthoreovirus causing goslings hepatitis. Vet Microbiol. (2018) 227:69–77. 10.1016/j.vetmic.2018.10.017 30473354

[20] Sterner F Rosenberger J Margolin A Ruff MJAd. *In vitro* and *in vivo* characterization of avian reoviruses. II. Clinical evaluation of chickens infected with two avian reovirus pathotypes. Avian Dis. (1989) 33:545–54. 10.2307/1591119 2549942

[21] Niu X Wang H Wei L Zhang M Yang J Chen H. Epidemiological investigation of H9 avian influenza virus, Newcastle disease virus, Tembusu virus, goose parvovirus and goose circovirus infection of geese in China. Transbound Emerg Dis. (2018) 65:e304–e16. 10.1111/tbed.12755 29134777

[22] Smither SJ Lear-Rooney C Biggins J Pettitt J Lever MS Olinger GG Jr. Comparison of the plaque assay and 50% tissue culture infectious dose assay as methods for measuring filovirus infectivity. J Virol Methods. (2013) 193:565–71. 10.1016/j.jviromet.2013.05.015 23748121

[23] Liu Q Zhang G Huang Y Ren G Chen L Gao J. Isolation and characterization of a reovirus causing spleen necrosis in Pekin ducklings. Vet Microbiol. (2011) 148:200–6. 10.1016/j.vetmic.2010.09.016 20970930

[24] Zhang Y Guo D Geng H Liu M Hu Q Wang J. Characterization of M-class genome segments of muscovy duck reovirus S14. Virus Res. (2007) 125:42–53. 10.1016/j.virusres.2006.12.004 17218035

[25] Zhu Y Li C Bi Z Chen Z Meng C Wang G. Molecular characterization of a novel reovirus isolated from Pekin ducklings in China. Arch Virol. (2015) 160:365–9. 10.1007/s00705-015-2513-0 25287130

[26] Xiao R Mi X Sun J Ding M Li C Zhu J. Interaction between Translocation-associated membrane protein 1 and σC protein of novel duck reovirus controls virus infectivity. Virus Genes. (2020) 56:347–53. 10.1007/s11262-020-01750-8 32180130

[27] Luo D Liu R Weng L Li K Qi X Gao Y. Genomic sequences and pathogenic characteristics of two variant duck reoviruses associated with spleen necrosis. Infect Genet Evol. (2021) 92:104847. 10.1016/j.meegid.2021.104847 33823307

[28] Zhang S Wang X Diao Y Tang Y. Recombinant characteristics, pathogenicity, and transmissibility of a variant goose orthoreovirus derived from inter-lineage recombination. Vet Microbiol. (2023) 277:109620. 10.1016/j.vetmic.2022.109620 36543090

[29] Smith S Gribble J Diller J Wiebe M Thoner T. Reovirus RNA recombination is sequence directed and generates internally deleted defective genome segments during passage. J Virol. (2021) 95:20. 10.1128/JVI.02181-20 33472930 PMC8103698

[30] Wang H Gao B Chen H Diao Y Tang Y. Isolation and characterization of a variant duck orthoreovirus causing spleen necrosis in Peking ducks, China. Transbound Emerg Dis. (2019) 66:2033–44. 10.1111/tbed.13252 31131546

[31] Yun T Hua J Ye W Yu B Chen L Ni Z. Comparative proteomic analysis revealed complex responses to classical/novel duck reovirus infections in Cairna moschata. Sci Rep. (2018) 8:10079. 10.1038/s41598-018-28499-3 29973707 PMC6031628

[32] Yu K Ti J Lu X Pan L Liu L Gao Y. Novel duck reovirus exhibits pathogenicity to specific pathogen-free chickens by the subcutaneous route. Sci Rep. (2021) 11:11769. 10.1038/s41598-021-90979-w 34083583 PMC8175558

[33] Czekaj H Kozdruń W Styś-Fijoł N Niczyporuk J Piekarska KJJovr. Occurrence of reovirus (ARV) infections in poultry flocks in Poland in 2010–2017. J Vet Res. (2018) 62:421–6. 10.2478/jvetres-2018-0079 30729197 PMC6364165

