# Supplementary material for: Characterization and pathogenicity evaluation of recombinant novel duck reovirus isolated from Southeast China
Source: Front Vet Sci. 2023 Mar 14;10:1124999. doi: 10.3389/fvets.2023.1124999 (PMC10043381; doi:10.3389/fvets.2023.1124999)
Supplement: Supplementary file 1 [file Data_Sheet_1.docx]

Supplementary Material

Characterization and pathogenicity evaluation of recombinant novel duck reovirus isolated from southeast China

**Huihu Yang^1^****^†^, Wandi Zhang^2†^,** **Meihong Wang^1^, Sheng Yuan^1^, Xuelian Zhang^1^, Feng Wen^1^, Jinyue Guo^1^, Kun Mei^1^,** **Shujian Huang^1*^,** **Zhili Li^1*^**

^1^College of Life Science and Engineering, Foshan University, Foshan 528231, Guangdong, China

^2^Nanyang Vocational College of Agriculture, Nanyang, China

**^†^**These authors contributed equally to this work and share first authorship

*** Correspondence:**Shujian Huang
[sjhuang.foshan@163.com](mailto:sjhuang.foshan@163.com)

Zhili Li

[pinganzhili@163.com](mailto:pinganzhili@163.com)

# Supplementary Tables

**Additional file 1**

Oligonucleotide primers used to amplify and sequence the L/S/M-class genes of Novel duck reovirus (NDRV) NDRV-ZSS-FJ20, NDRV-LRS-GD20 and NDRV-FJ19.

| **Primers** | **Oligonucleotide sequence（5’➞3’）** | **Length of segments（bp）** |
| --- | --- | --- |
| L1a-F | GCTTTTTCTCCGAACGCCGA | 2041 |
| L1a-R | TAGGGTCATCCATAGGCAAATTCTC |  |
| L1b-F | CCTATGGATGACCCTAACTT | 1934 |
| L1b-R | GATGAATAACCTCCAACGA |  |
| L2a-F | GCTTTTTCCTCACCATGCAT | 1958 |
| L2a-R | TGACACATAACCTGGAAACC |  |
| L2b-F | GTCCTCAATGCCTATTTCCG | 1913 |
| L2b-R | GATGAGTAATTCCTCGAGCCA |  |
| L3a-F | GCTTTTACACCCATGGCTCA | 2118 |
| L3a-R | AGTGGGTCGTCCAGCGTAA |  |
| L3b-F | CTTTCAATCCCTCCGCTG | 1921 |
| L3b-R | GATGAGTAACACCCTTCTACTGGAG |  |
| M1-F | GCTTTTCTCGACATGGCCTATCTAGC | 2284 |
| M1-R | GATGAATATCTCAAGACGGCTAACCCAGG |  |
| M2-F | GCTTTTTGAGTGCTAACCT | 2158 |
| M2-R | GATGAGTAACGTGCTAACC |  |
| M3-F | GCTTTTTGAGTCCTAGCGTGG | 1996 |
| M3-R | GATGAGTAACCGAGTCCGCCGTGG |  |
| S1-F | GCTTTTTTCTTCTCTGCCCAT | 1568 |
| S1-R | GATGAATAGCTCTTCTCATCGTGC |  |
| S2-F | GCTTTTTCTCCCACGATGGC | 1324 |
| S2-R | GATGAATACACCCACGCGCTAC |  |
| S3-F | GCTTTTTGAGTCCTCAGCGTG | 1202 |
| S3-R | GATGAATAGGCGAGTCCCGC |  |
| S4-F | GCTTTTTGAGTCCTTGTGCA | 1191 |
| S4-R | GATGAATAAGAGTCCAAGTCGC |  |

**Additional file 2**

details of data of the growth kinetics

| **Hours post infection (h)** | **Virus titer (LogTCID50/0.1mL) (mean±SD)** | | |
| --- | --- | --- | --- |
|  | **Vero cells** | **BHK-21 cells** | **LHM cells** |
| 12 | 1.3±0.1 | 1.6±0.05 | 1.6±0.1 |
| 24 | 1.7±0.15 | 2.2±0.15 | 2.3±0.05 |
| 36 | 2.5±0.2 | 2.4 | 3.3±0.1 |
| 48 | 3.1±0.15 | 3.1 | 4.4±0.25 |
| 60 | 3.6 | 4.3±0.1 | 5.5±0.35 |
| 72 | 4.2 | 5.2±0.05 | 5.6±0.3 |
| 84 | 4.2±0.15 | 5.3±0.1 | 5.8±0.1 |
| 96 | 4.3±0.2 | 5.3±0.2 | 5.6±0.2 |
| 108 | 3.9 | 5.3±0.1 | 5.5±0.1 |
| 120 | 3.7±0.15 | 5.1±0.05 | 5.5±0.1 |

**Additional file 3**

GenBank ID of sequences

used in this study.

| **Virus** | **Strain** | **λA（L1）** | **λB（L2）** | **λC（L3）** | **μA（M1）** | **μB（M2）** | **μNS（M3）** | **p10/p18/σC（S1）** | **σA（S2）** | **σB（S3）** | **σNS（S4）** | **Origin** | **Host** | **Time** |
| --- | --- | --- | --- | --- | --- | --- | --- | --- | --- | --- | --- | --- | --- | --- |
| NDRV | SH12 | MH510245 | MH510246 | MH510247 | MH510248 | MH510249 | MH510250 | MH510251 | MH510252 | MH510253 | MH510254 | China | Muscovy duck | 2012 |
|  | DH13 | MH510255 | MH510256 | MH510257 | MH510258 | MH510259 | MH510260 | MH510261 | MH510262 | MH510263 | MH510264 | China | Muscovy duck | 2013 |
|  | 091 | JX478250 | JX478251 | JX478252 | JX478253 | JX478254 | JX478255 | JX478256 | JX478257 | JX478258 | JX478259 | China | Pekin duck | 2009 |
|  | NP03 | KC312700 | KC312701 | KC312702 | JF320802 | JF320801 | JF320800 | KC312699 | JF320803 | GQ888710 | GU338025 | China | Muscovy duck | 2009 |
|  | HN5d | KT861587 | KT861588 | KT861589 | KT861590 | KT861591 | KT861592 | KT861593 | KT861594 | KT861595 | KT861596 | China | Pekin duck | 2013 |
|  | SD-12 | KJ879924 | KJ879925 | KJ879926 | KJ879927 | KJ879928 | KJ879929 | KJ879930 | KJ879931 | KJ879932 | KJ879933 | China | Wild Mallard Duck | 2014 |
|  | J18 | JX478260 | JX478261 | JX478262 | JX478263 | JX478264 | JX478265 | JX478266 | JX478267 | JX478268 | JX478269 | China | Muscovy duck | 2008 |
|  | 03G | JX145333 | JX145329 | JX145330 | JX145331 | JX145332 | JX145333 | JX145334 | JX145336 | JX145336 | JX145337 | China | Goose | 2003 |
|  | ZJ00M | KF154110 | KF154111 | KF154112 | KF154113 | KF154114 | KF154115 | KF154116 | KF154117 | KF154118 | KF154119 | China | Muscovy duck | 2009 |
|  | TH11 | KC493572 | KC493573 | KC493574 | JX440513 | JX440514 | JX440512 | KC493571 | JQ664689 | JX826588 | JX826589 | China | Pekin duck | 2011 |
| MDRV | 815-12 | KC508647 | KC508648 | KC508649 | KC508650 | KC508651 | KC508652 | KC508656 （S4） | KC508653  （S1） | KC508654（S2） | KC50865（S3） | China | Muscovy duck | 2010 |
|  | ZJ200M | KF306082 | KF306083 | KF306084 | KF306085 | KF306086 | KF306087 | KF306091（S4） | KF306088（S1） | KF306089（S2） | KF306091（S3） | China | Muscovy duck | 2011 |
|  | D1546 | KJ871017 | KJ871019 | KJ871018 | KJ871020 | KJ871021 | KJ871022 | KJ871023 | KJ871024 | KJ871025 | KJ871026 | France | Muscovy duck | 2010 |
|  | D2044 | KJ871007 | KJ871009 | KJ871008 | KJ871010 | KJ871011 | KJ871012 | KJ871013 | KJ871014 | KJ871015 | KJ871016 | France | Muscovy duck | 2012 |
| Chicken  ARV | 1733 | KF741706 | KF741707 | KF741708 | KF741709 | KF741710 | KF741711 | KF741712 | KF741713 | KF741714 | KF741715 | China | Chicken | 2013 |
|  | AVS-B | FR694191 | FR694192 | FR694193 | FR694194 | FR694195 | FR694196 | FR694197 | FR694198 | FR694199 | FR694200 | USA | Chicken | 2010 |
|  | D1007 | KR476798 | KR476799 | KR476800 | KR476801 | KR476802 | KR476803 | KR476804 | KR476805 | KR476806 | KR476807 | Hungary | quail | 2015 |
|  | T1781 | KC865786 | KC865787 | KC865788 | KC865789 | KC865790 | KC865791 | KC865792 | KC865793 | KC865794 | KC865795 | Hungary | Chicken | 2012 |
|  | S1133 | KF741756 | KF741757 | KF741758 | KF741759 | KF741760 | KF741761 | KF741762 | KF741763 | KF741764 | KF741765 | USA | Chicken | 2004 |
|  | T-98 | EU616739 | JN641889 | EU616738 | EU616736 | EU616742 | EU616743 | EF057398 | JN641887 | EF030499 | JN641884 | China | Chicken | 2008 |
| MRV | MRV-2018 | MW929746 | MW929747 | MW929748 | MW929749 | MW929750 | MW929751 | MW929755  （S4） | MW929752  （S1） | MW929753  （S2） | MW929754  （S3） | USA | Porcine | 2018 |
|  | MRV00304 | KJ676379 | KJ676380 | KJ676381 | KJ676382 | KJ676383 | KJ676384 | KJ676388 | KJ676385 | KJ676386 | KJ676387 | USA | Bovine | 2014 |
|  | SHR-A | JX415465 | JX415466 | JX415467 | JX415468 | JX415469 | JX415470 | JX415474 | JX415471 | JX415472 | JX415473 | China | Porcine | 2011 |

**Additional file 4**

Sequence identities (%) of nt and aa between NDRV-ZSS-FJ20，NDRV-LRS-GD20 and NDRV-FJ19 and other NDRV, MDRV and chicken ARV

| **Gene/**  **Protein** | **NDRV-ZSS-FJ20**  **VS NDRV-LRS-GD20**  **(nt/aa)** | **NDRV-ZSS-FJ20 VS**  **NDRV-FJ19**  **(nt/aa)** | **NDRV-FJ19**  **VS NDRV-LRS-GD20**  **(nt/aa)** | **NDRV-ZSS-FJ20**  **VS**  **NDRV**  **(nt/aa)** | **NDRV-ZSS-FJ20**  **VS**  **MDRV**  **(nt/aa)** | **NDRV-ZSS-FJ20**  **VS**  **chicken ARV**  **(nt/aa)** | **NDRV-LRS-GD20**  **VS**  **NDRV**  **(nt/aa)** | **NDRV-LRS-GD20**  **VS**  **MDRV**  **(nt/aa)** | **NDRV-LRS-GD20**  **VS**  **chicken ARV**  **(nt/aa)** | **NDRV-FJ19**  **VS**  **NDRV**  **(nt/aa)** | **NDRV-FJ19**  **VS**  **MDRV**  **(nt/aa)** | **NDRV-FJ19**  **VS**  **chicken ARV**  **(nt/aa)** |
| --- | --- | --- | --- | --- | --- | --- | --- | --- | --- | --- | --- | --- |
| L1 | 97.2 /99.7 | 87.1 /97.6 | 86.7 /97.0 | 86.7-98.6 /98.2-99.9 | 86.1-86.7 /96.8-97.8 | 78.0-78.8 /93.5-95.4 | 86.5-98.2 /97.9-99.6 | 86.1-86.5 /95.6-96.9 | 77.6-78.5 /91.5-94.8 | 87.1-99.9 /98.1-100 | 87.8-89.5 /94.8-96.0 | 78.8-79.2 /90.6-94.1 |
| L2 | 98.4 /99.6 | 98.3 /99.1 | 98.2 /99.2 | 88.1-98.5 /97.8-99.5 | 88.0-88.6 /96.5-97.9 | 76.9-77.8 /90.1-92.3 | 87.7-98.5 /98.5-99.4 | 87.8-88.3 /96.3-97.0 | 76.8-77.7 /91.0-91.9 | 88.4-99.6 /98.9-99.9 | 88.3-89.7 /95.9-96.4 | 76.8-77.9 /91.2-92.0 |
| L3 | 98.0 /99.2 | 95.9 /98.6 | 95.4 /98.8 | 95.5-98.0 /96.8-99.3 | 79.9-80.9 /91.9-94.6 | 70.4-71.2 /78.9-80.1 | 95.9-98.5 /97.8-99.7 | 80.0-81.4 /91.5-95.0 | 70.9-71.2 /77.4-79.3 | 96.2-98.1 /98.5-99.8 | 79.0-80.1 /90.8-94.9 | 70.1-70.3 /78.1-79.0 |
| M1 | 97.1 /98.9 | 96.7 /98.6 | 96.1 /97.9 | 95.1-97.4 /96.9-99.5 | 80.9-82.2 /91.3-97.4 | 73.5-75.6 /85.1-88.3 | 95.1-97.6 /97.1-99.7 | 81.1-82.1 /91.0-96.5 | 73.3-76.2 /84.1-85.9 | 96.2-99.5 /98.9-99.9 | 81.4-82.3 /91.6-95.4 | 73.6-75.9 /83.6-85.0 |
| M2 | 97.8 /99.0 | 98.1 /98.9 | 97.5 /99.5 | 88.1-98.3 /95.8-99.6 | 67.1-67.3 /74.3-78.6 | 71.2-77.9 /86.3-89.4 | 87.7-98.3 /96.1-99.1 | 67.3-68.6 /75.2-76.1 | 71.5-78.6 /86.4-88.5 | 88.2-99.6 /97.9-99.6 | 67.3-68.8 /74.5-75.6 | 71.5-78.4 /85.4-87.3 |
| M3 | 99.1 /99.8 | 96.7 /97.9 | 96.7 /99.5 | 94.2-99.5 /96.1-98.8 | 86.2-88.5 /91.0-94.5 | 75.1-76.0 /77.4-79.2 | 94.6-99.2 /96.9-99.9 | 86.3-88.7 /90.2-93.9 | 75.0-76.8 /78.1-79.0 | 95.7-99.9 /97.8-100 | 87.5-88.4 /90.3-92.1 | 75.3-76.1 /78.3-79.0 |
| σC | 96.0 /98.5 | 98.0 /99.0 | 94.5 /98.7 | 91.9-98.5 /94.6-98.8 | 38.1-39.6 /36.5-38.2 | 38.9-39.9 /26.9-28.0 | 91.9-97.1 /94.3-97.9 | 38.8-39.1 /35.4-38.9 | 39.0-40.1 /27.3-28.5 | 93.1-99.7 /95.1-98.1 | 37.6-39.5 /36.7-39.1 | 38.9-40.5 /26.9-28.1 |
| σA | 97.5 /99.2 | 88.1 /97.3 | 88.0 /97.0 | 84.8-98.3 /96.8-99.8 | 88.4-94.3 /95.1-98.9 | 77.3-80.4 /89.3-91.0 | 84.9-99.8 /98.9-99.9 | 88.8-95.0 /95.3-97.6 | 77.2-80.3 /88.8-89.7 | 89.8-99.7 /99.1-99.8 | 88.9-95.0 /95.3-96.2 | 77.7-80.9 /88.1-88.5 |
| σB | 98.8 /99.6 | 96.0 /97.9 | 96.3 /99.8 | 94.1-98.7 /95.1-99.7 | 71.1-72.8 /66.7-71.2 | 69.9-71.3 /69.9-70.3 | 94.3-98.9 /94.8-99.8 | 71.2-71.6 /65.3-70.0 | 69.2-71.5 /70.1-70.8 | 95.8-99.9 /93.8-100 | 71.7-72.5 /62.1-67.2 | 67.9-71.8 /68.8-69.8 |
| σNS | 99.1 /99.9 | 96.8 /98.5 | 96.8 /98.9 | 93.4-99.8 /97.7-99.6 | 83.1-85.6 /94.0-97.6 | 78.3-79.8 /90.2-90.9 | 92.8-99.1 /98.4-99.9 | 83.2-85.6 /93.6-96.8 | 79.2-80.0 /89.5-91.0 | 93.2-99.7 /99.2-99.9 | 84.1-86.5 /92.8-95.4 | 79.2-80.1 /89.2-90.8 |

NDRV strains :SD-12, MDRV-SH12, MDRV-DH13, 03G, 091, TH11, NP03, J18, ZJ00M and HN5d

MDRV strains : ZJ2000M, D1546, D2044 and 815-12

chicken ARV strains :1733, S1133, T98, AVS-B, D1007 and T1781

nt, nucleotide sequence

aa, amino acid sequence
